# Identification of insect-specific target genes for development of RNAi based control of the *Eucalyptus* gall pest *Leptocybe invasa* Fisher & La Salle (Hymenoptera: Eulophidae)

**DOI:** 10.1186/1753-6561-5-S7-P98

**Published:** 2011-09-13

**Authors:** Mathish Nambiar-Veetil, Manoharan Sangeetha, K S Sowmiya Rani, V Aravinthakumar, R K Selvakesavan, Aiyar Balasubramanian, R Venkatachalam, Abraham Sanu Mary, John Prasanth Jacob, N Krishna Kumar

**Affiliations:** 1 Institute of Forest Genetics and Tree Breeding, Coimbatore, India

## Background

*Eucalyptus* is grown in around 3.9 Mha in India. *Leptocybe invasa* Fisher & La Salle (Hymenoptera: Eulophidae) has emerged as a serious pest in *Eucalyptus* causing considerable loss of quality planting materials besides loss in productivity and quality of timber. Current strategies for its control include selected deployment of *Eucalyptus* clones tolerant to the pest resulting in several productive *Eucalyptus* genetic resources, not being considered for plantation programmes, as in the case of the widely planted *Eucalyptus* clone, ITC 10, and ITC 271. The securely ensconced grub within the gall for a period of around 4 months post oviposition makes it refractory to pesticide applications. This feature, however, makes plant- incorporated protectants, via RNAi approaches a potential strategy for engineering resistance [1-3]. However, application of RNAi technology requires determination of sequence information of insect-specific genes so that off-target effects in plants as well as human beings are avoided.

## Methods

Grubs were collected from the galls of *E. camaldulensis* clones, APNP 1.1 and ITC 351, infested with *L .invasa* from the nursery of the Institute of Forest Genetics and Tree Breeding, Coimbatore, India. Genomic DNA was isolated from the grubs homogenized in liquid nitrogen using a modified CTAB protocol [4]. RNA was isolated from the grubs using Qiagen RNeasy plant mini kit, followed by cDNA synthesis using SmartScribe reverse transcriptase.

Grubs were also directly used for PCR amplification circumventing DNA isolation. Furthermore, to enable multiple PCR analysis, a single grub was collected into 50 µl of sterile distilled water, vigorously vortexed for 2 min followed by brief denaturation at 95^0^C for 10 min and centrifugation at 10,000 rpm for 10 min. The supernatant was used for PCR analysis.

The PCR mix consisted either 100 ng genomic DNA or 100 ng cDNA or a single grub or 2 µl of supernatant from denatured grub, in 1X PCR buffer, 0.8 mM dNTPs, 1 µM of each primer, 2.5 U *Pfu* polymerase (Fermentas). The PCR conditions used were initial denaturation at 94^0^C for 5 min, cycle denaturation at 94^0^C for 40s; annealing at 60^0^C for 40s; extension at 72^0^C for 2 min for 30 cycles, and a final extension of 72^0^C for 5 min. In case of grub PCR, initial denaturation was for 10 min at 95^0^C. The PCR products were resolved on a 1.0 % Agarose gel in 1X TAE. When multiple bands were obtained, the products were gel eluted and either directly sequenced or cloned into pGEMTeasy vector prior to sequencing. Sequencing was done using the ABI PRISM 3130 XL Genetic Analyzer using Big Dye Terminator version 3.1” Cycle sequencing kit through the commercial sequencing services available at Chromous Biotech India Pvt Ltd.

## Results and discussion

Chitin synthase gene was partially amplified using the degenerate primer sets, CSF3 (5'-TGYGCGACHA TGTGGCACGARAC-3')/ CSR1 (5'-GTCCTCSCCYT GRTCGTAYTGCAC-3') and CSF1 (5'-YTGGAY GGMGACATMGAYTTC-3')/ CSR1 (5'- GTCCTCSCCYTGRTCGTAYTGCAC-3') [5] from genomic DNA and cDNA. CSF3/R1 primers yielded multiple bands of which the expected 1KB size amplicon was sequenced. CSF1/R1 primers yielded an amplicon of 370 bp, which overlapped with the sequence from CSF3/R1. The sequence data obtained were aligned, annotated, and a sequence of 624 bp was submitted to NCBI (Accession number: JF772551/to be published). The sequence comprising 2 exons and 2 introns, corresponded to 138 amino acids on conceptual translation. BLAST analysis of the nucleotide sequence showed 85 % homology to *Nasonia vitripennis* chitin synthase 1 and 76 % to *Apis mellifera* krotzkopf verkehrt (chitin synthase1) sequences. Furthermore, a rapid method of PCR amplification directly from grubs was developed. The amplicons generated using the primers CSF1/CSR1 yielded the same sequence, indicating its utility for rapid PCR screening of *L. invasa* grubs. In order to identify housekeeping genes for use as reference genes during quantitative PCR analysis, Elongation factor-1 alpha (EF-1 alpha) was partially amplified using primers EF1-F (5'-GGTATCGACAAA CGT ACCATCG-3') /EF1-R (5'- AATCGAGCACAGG TGTGTAACC-3') and the sequence data of 534 bp was submitted to NCBI (Accession number: JF772552/to be published). Blast analysis of this sequence showed 91 % similarity to *N. vitripennis* EF-1 alpha.

**Figure 1:**
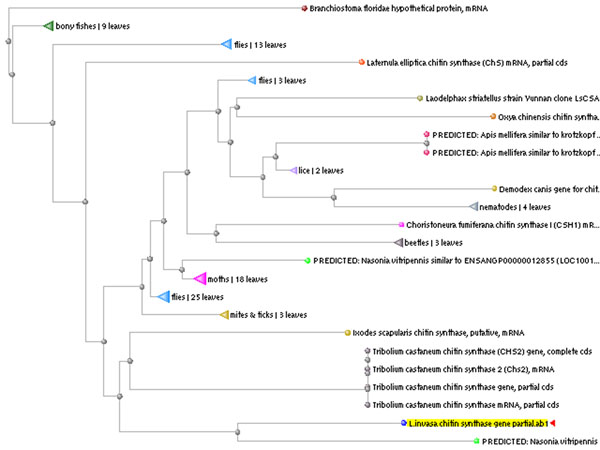
Tree view produced using BLAST pairwise alignment for the partial sequence of *L. invasa* chitin synthase1 gene

## Conclusion

Basic techniques like nucleic acid isolation and PCR amplification were adapted for this less studied insect pest*.* A rapid method for PCR amplification directly from the insect grub was developed for use in *L. invasa.* Our efforts have unraveled the partial gene sequence of *L. invasa* chitin synthase, a processive enzyme involved in chitin biosynthesis during different stages of insect development. The sequence of chitin synthase and EF-1 alpha represent the first genome sequence information for *L. invasa.* Chitin synthase offers a potential target for RNAi based pest control owing to its crucial role in growth and development of insect. Following full length sequence determination of *L. invasa* chitin synthase gene, RNAi target regions will be chosen for development of RNAi constructs. EF-1 alpha will be used as the reference gene in RT-PCR studies to quantitate the effect of RNAi on the transcript levels of the target gene. The effect of *Eucalyptus*-expressed dsRNA molecules cognate for chitin synthase, will be evaluated on the growth and development of *L. invasa.* Towards this end, protocols have been optimized for generation of transgenic *Eucalyptus*[6].

## References

[B1] Baum JA Bogaert T Clinton W Heck GR Feldmann P Ilagan O Johnson S Plaetinck G Munyikwa T Pleau M Vaughn T Roberts J Control of coleopteran insect pests through RNA interference Nat Biotechnol 2007 25 11 1322 6 10.1038/nbt1359 17982443

[B2] Mao YB Cai WJ Wang JW Hong GJ Tao XY Wang LJ Huang YP Chen XY Silencing a cotton bollworm P450 monooxygenase gene by plant-mediated RNAi impairs larval tolerance of gossypol Nat Biotechnol 2007 25 11 1307 13 10.1038/nbt1352 17982444

[B3] Mao YB Tao XY Xue XY Wang LJ Chen XY Cotton plants expressing CYP6AE14 double-stranded RNA show enhanced resistance to bollworms Transgenic Res 2010 DOI10.1007/s11248-010-9450-1 10.1007/s11248-010-9450-1 PMC3090577 20953975

[B4] Chen H Rangasamy M Tan SY Wang H Siegfried BD Evaluation of Five Methods for Total DNA Extraction from Western Corn Rootworm Beetles PLoS one 2010 5 8 e11963 10.1371/journal.pone.0011963 20730102 PMC2921343

[B5] Kumar N.S Tang B Chen X Tian H Zhang W Molecular cloning, expression pattern and comparative analysis of chitin synthase gene B in Spodoptera exigua Comp Biochem Physiol B 2008 149 447 453 10.1016/j.cbpb.2007.11.005 18178495

[B6] Mathish NV Lalitha S Sudha N Brindha D Prashant K Balasubramanian A Sumathi R Karthikeyan C Sharadha N Shanthi A Sivakumar V Yasodha R Suryaprakash M Franche C Gherbi H Swistoonoff S Florence A Hocher V Bogusz D Blumwald E Gurumurthi K Optimization of genetic transformation methods in Eucalyptus: towards understanding and enhancing salt tolerance National Conference on Frontiers in Plant Molecular Biology 2010 Bharathidasan University, Tiruchirappalli

